# Effects of motor stimulation of the tibial nerve on corticospinal excitability of abductor hallucis and pelvic floor muscles

**DOI:** 10.3389/fresc.2022.1089223

**Published:** 2023-01-16

**Authors:** Gevorg Eginyan, Xueqing Zhou, Alison M. M. Williams, Tania Lam

**Affiliations:** ^1^ International Collaboration on Repair Discoveries (ICORD), Faculty of Medicine, University of British Columbia (UBC), Vancouver, BC, Canada; ^2^ School of Kinesiology, University of British Columbia (UBC), Vancouver, BC, Canada

**Keywords:** tibial nerve stimulation, pelvic floor muscles, abductor hallucis, corticospinal excitability, continuous stimulation, intermittent stimulation

## Abstract

**Introduction:**

Peripheral nerve stimulation can modulate the excitability of corticospinal pathways of muscles in the upper and lower limbs. Further, the pattern of peripheral nerve stimulation (continuous vs. intermittent) may be an important factor determining the modulation of this corticospinal excitability. The pelvic floor muscles (PFM) are crucial for maintaining urinary continence in humans, and share spinal segmental innervation with the tibial nerve. We explored the idea of whether the neuromodulatory effects of tibial nerve stimulation (TibNS) could induce effects on somatic pathways to the PFM. We evaluated the effects of two patterns of stimulation (intermittent vs. continuous) on corticospinal excitability of the PFM compared to its effect on the abductor hallucis (AH) muscle (which is directly innervated by the tibial nerve). We hypothesized that intermittent TibNS would increase, while continuous stimulation would decrease, the excitability of both AH and PFM.

**Methods:**

Twenty able-bodied adults (20-33 years of age) enrolled in this study. TibNS was delivered either intermittently (1 ms pulses delivered at 30Hz with an on:off duty cycle of 600:400 ms, for 60 min), or continuously (1 ms pulses delivered at 30Hz for 36 min) just above the motor threshold of the AH. We randomized the order of the stimulation pattern and tested them on separate days. We used surface electromyography (EMG) to record motor-evoked responses (MEP) in the PFM and AH following transcranial magnetic stimulation (TMS). We generated stimulus-response (SR) curves to quantify the changes in peak-to-peak MEP amplitude relative to TMS intensity to assess changes in corticospinal excitability pre- and post-stimulation.

**Results and Conclusion:**

We found that TibNS increased corticospinal excitability only to AH, with no effects in PFM. There was no difference in responses to continuous vs. intermittent stimulation. Our results indicate a lack of effect of TibNS on descending somatic pathways to the PFM, but further investigation is required to explore other stimulation parameters and whether neuromodulatory effects may be spinal in origin.

## Introduction

Peripheral nerve stimulation can induce transient as well as longer-lasting changes to the excitability of both sensory and motor areas of the human cortex (1–4). The proposed mechanism of this cross-system plasticity might be attributed to sensorimotor integration, facilitating communication between the somatosensory system and motor system. The potential for peripheral nerve stimulation to modulate both the sensory and motor systems supports the use of this technique for diverse clinical applications, from restoration of upper or lower limb motor function (5–8) to managing dysphagia (9), or the symptoms of lower urinary tract dysfunction (10, 11).

Multiple studies have shown that peripheral nerve stimulation can modulate excitability along descending pathways from the motor cortex (12). For example, previous findings indicate that a single session of electrical stimulation of a peripheral mixed nerve in the hand results in increased excitability of the corticospinal pathways and size of the primary motor cortical representation of muscles innervated by that same nerve (1, 2, 13). Furthermore, various aspects of peripheral nerve stimulation parameters, including stimulation frequency (4, 14, 15), intensity (3, 4), duration (16, 17), and even pattern of current delivery (18) seem to affect the plasticity of corticospinal projections differently. Stimulus intensities above motor threshold appear to be important for modulating corticospinal excitability, and the stimulus duration seems to correspond to the maintenance of effects, but the effect of stimulation frequency or pulse duration appears less clear (12). There is also some indication that an intermittent pattern of stimulation (with an on-off duty cycle) increases corticospinal excitability, while a continuous pattern suppresses corticospinal excitability (18).

Much of our understanding of the neuromodulatory effects of peripheral nerve stimulation is based on studies of the upper limb, with fewer studies of the lower limb. In the upper limb, it appears that neuromodulatory effects are focal, whereby modulation is observed in corticospinal projections to the target muscle alone (1, 2, 19). But in the lower limb, effects on corticospinal excitability may be more global; there is evidence that stimulation of a peripheral nerve in the leg modulates corticospinal projections not only to the target muscle, but also to other muscles in the lower limb innervated by different nerves (19). Such distributed effects of peripheral nerve stimulation in the lower limb may reflect the need for integrated activity of the lower limb muscles, along with muscles across the body, to perform gross motor functions such as walking and balance.

The pelvic floor muscles (PFM) are a critical muscle group needed to maintain the position of the pelvic organs against changes in intra-abdominal pressure that accompany different motor tasks, including those involving the postural, respiratory, or locomotor systems (20–25). PFM activity varies with different tasks involving the lower limbs, namely standing (compared to supine lying) (26), walking (22, 23, 25, 27), and jumping (28), raising the question of whether corticospinal inputs to these muscles may also be modulated by lower limb peripheral nerve stimulation. The PFM are innervated by somatic sacral nerves originating from S2 to S4, sharing spinal segmental innervation with the tibial nerve (L4-S3). The PFM and tibial nerve also both happen to be clinical targets for bladder management. The PFM are an important target of physiotherapy interventions for managing lower urinary track dysfunction, given this muscle group's crucial role in maintaining continence (29, 30), while the tibial nerve at the medial malleolus is a target for peripheral nerve stimulation therapies for idiopathic and neurogenic lower urinary tract dysfunction (10, 11). Tibial nerve stimulation (TibNS) was introduced decades ago (31) and is reminiscent of traditional acupuncture techniques to treat overactive bladder (32). Considering that the tibial nerve shares segmental innervation with the sacral autonomic and somatic nerves that innervate the bladder and external sphincter, it has been surmised that TibNS may be involved in modulating voiding reflex pathways through cross-signaling mechanisms (10, 32).

The finding that stimulation of a lower limb peripheral nerve could enable diffuse effects altering corticospinal excitability of both target and non-target muscles, along with the notion that there is a cross-signaling mechanism between TibNS and the nerves serving the lower urinary tract through their shared segmental innervation (10, 32), raises the possibility of a widespread effect of afferent input from the lower limb to somatic pathways involved in multiple systems. In this study, we sought to examine this question by investigating the effects of TibNS on the corticospinal excitability of the PFM (which shares segmental innervation with the tibial nerve) compared to its effect on the abductor hallucis muscle (which is directly innervated by the tibial nerve). Because the clinical parameters used in TibNS employ continuous patterns of stimulation (10, 11), but physiological studies of the effects of peripheral nerve stimulation on corticospinal excitability employ intermittent patterns, we also sought to compare the effects of continuous vs. intermittent patterns of TibNS on corticospinal excitability. We hypothesized that the corticospinal excitability will be acutely affected in both abductor hallucis (AH) and PFM following 1 h of TibNS and that an intermittent pattern of TibNS will increase, but a continuous stimulation pattern will decrease, corticospinal excitability in both AH and the PFM.

## Materials and methods

### Participants

Twenty able-bodied individuals (10 females and 10 males) between 20 and 33 years of age with a mean height of 170 cm (SD 10 cm) and mass of 65 kg (SD 13 kg) were recruited for this study. Participants were excluded from participation if they had been diagnosed with any form of urinary incontinence, detrusor overactivity, overactive/neurogenic bladder syndrome, pelvic pain, PFM dysfunction, or any other musculoskeletal and/or neurological impairment; had been pregnant, given birth, or had any urogenital/abdominal surgery within the last 12 months; or were experiencing acute genital and/or bladder infection or menses at the time of the participation. Participants were also screened for transcranial magnetic stimulation (TMS) contraindications and precautions including presence of any permanent metal fixtures within the cranium (excluding dental fillings), or any other parts of the body; history of seizures/epilepsy, or taking medication that lower seizure threshold; history of cranial and/or brain surgery or trauma; presence of psychiatric disorder or taking any psychedelic medications; and presence of electrodes implanted within the central or peripheral nervous systems.

Study procedures were approved by the University of British Columbia Clinical Research Ethics Board (H20-02749) and all participants provided written informed consent.

### Experimental design

This study employed a single-blinded cross-sectional repeated-measures pre-test-post-test crossover design to compare the effects of intermittent and continuous TibNS on corticospinal excitability of the AH and PFM.

Each participant visited the laboratory for two separate testing sessions separated by at least three days, in which an intermittent pattern of TibNS was applied on one occasion and continuous TibNS was applied on the other occasion. Visits were separated by ≤2 weeks. The order of testing sessions was randomized and counterbalanced across all participants.

### Electromyography setup

We recorded surface EMG from both PFM and AH (Trigno, Delsys Inc, Boston, USA). We recorded EMG from the PFM using a pair of disposable disc surface electrodes affixed approximately 1 cm lateral from the anus, bilaterally. The disposable electrodes were then connected to a wireless Trigno Snap-Lead EMG sensor *via* a custom-made connector. For AH EMG recordings, we affixed a wireless Trigno Mini sensor on the skin overlying the AH muscle belly on the right foot. We also placed two other surface EMG electrodes over the right rectus abdominus and gluteus maximus muscles to verify that the Kegel contractions were performed in isolation without any accompanying abdominal and/or gluteal contractions.

We sampled all EMG signals at 2,000 Hz and streamed them into a custom-designed data acquisition system (LabView National Instruments, Austin, TX, USA), which allowed for an on-line visualization and monitoring of the signals. The Trigno system has a built-in amplification (x909) and band-pass filter (20–450 Hz).

### TMS and hotspot searching

We recorded motor-evoked potentials (MEPs) in the PFM and AH muscles using TMS applied with a 110-cm double cone coil (Magstim 200 stimulator; MagStim Company Ltd, Dyfed, UK). To ensure the consistency of coil position and orientation, we used a custom-designed TMS navigation tool using the Optotrak 3D motion capture system (Northern Digital Inc, Waterloo, Canada) and Unity (Unity Technologies, San Francisco, USA), a software designed to create and operate 3D virtual reality components. We affixed a rigid body consisting of 3 infrared-emitting diodes on the participant's forehead with a head strap, and taped an identical rigid body directly onto the double cone coil. Using NDI First Principles software (Northern Digital Inc, Waterloo, Canada), we digitized additional virtual landmarks on the participant's head: the right and left preauricular points, nasion (depressed area between the eyes), inion (occipital protuberance), mental protuberance of the mandible, and the vertex. Following the guidelines of the international 10–20 system for EEG electrode placement, vertex was defined as the halfway point of the line measured between the nasion to inion (33). We digitized an additional virtual landmark on the central aspect of the inside surface of the double-cone coil.

We streamed data from the virtually-digitized landmarks and rigid-bodies in real-time into the custom-designed navigation program in Unity. The navigation program provided the examiner with real-time 3D feedback about the position and orientation of the coil in relation to the reference point on the scalp for TMS application (hot spot).

Prior to acquiring a baseline measure of corticospinal excitability, we determined the optimal position and orientation of the TMS coil for both PFM and AH. These hot spot locations were defined as the optimal scalp sites to evoke 5 consistent MEPs in the target muscle (AH or PFM) of the highest amplitude and shortest latency at the lowest stimulation intensity (34). Once established, we marked the optimal scalp sites (1 for AH and 1 for PFM) relative to each participant's vertex and saved them in the navigation system. The same hot spot locations were then used throughout the data collection session pre- and post-TibNS.

### Experimental protocol

Participants sat comfortably in a height-adjustable reclining chair with their knees flexed at 90 degrees and feet planted on the floor. To determine the average amount of voluntary activity that participants could generate in AH and PFM, we asked them to perform 3 trials of attempted maximal contractions of the PFM (35) followed by 3 separate trials of AH contractions. We calculated the average EMG amplitude of the attempted maximal contractions for each muscle over the three trials and used this value to standardize the level of background muscle contraction during the TMS trials.

During each visit, we determined the baseline level of corticospinal excitability through MEP recordings elicited by TMS over the primary motor cortical areas of PFM and AH. We instructed participants to maintain a background muscle contraction of ∼10% of their average attempted maximal contraction during each TMS trial. To help participants maintain a steady contraction, we displayed the real-time rectified and filtered EMG signals on a computer screen for visual biofeedback. We asked each participant to contract their PFM and AH muscles until the rectified EMG signal reached a horizontal line on the screen corresponding to ∼10% of their attempted maximal contraction. This slight background contraction enhanced our ability to evoke MEPs in the target muscles and reduced MEP variability across trials (36).

We delivered the TMS in a series of blocks, each consisting of 7 single-pulse TMS stimuli over the hot spot locations of each muscle ranging from TMS intensities below the active motor threshold until the maximum stimulation output (MSO). We used increasing stimulation intensity in increments of ∼5% MSO in order to generate stimulus-response (SR) curves for each muscle separately (further details of SR curves described below). Blocks at each stimulus intensity were presented in randomized order, but the order was the same between pre- and post-TibNS.

Following the baseline measures of corticospinal excitability of PFM and AH, participants underwent either intermittent TibNS or continuous TibNS (based on random assignment). The examiner performing TMS was blinded to the type of stimulation pattern participants received. To avoid any potential confounding of order effects, we also randomized the order of TMS assessments for each muscle and counterbalanced across all participants. Immediately after the TibNS, we obtained another set of SR curves from the target muscles.

### Tibial nerve stimulation

**Figure 1:**
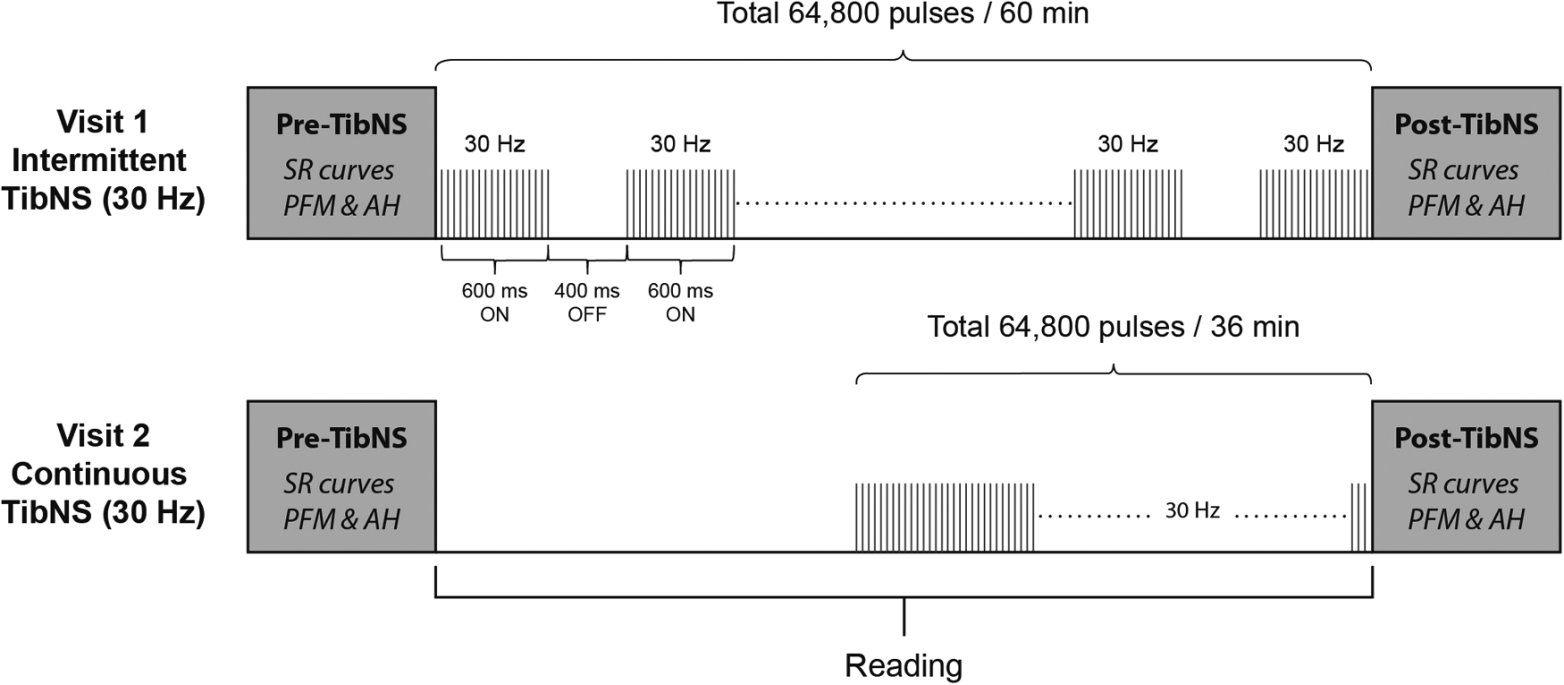
Schematic of the study protocol and TibNS interventions. Intermittent TibNS (30** **Hz), continuous TibNS (30** **Hz) with a total of 64,800 pulses were applied. The pulse amplitudes were equalized between these stimulation parameters, and pulse width duration set at 1** **ms.

TibNS parameters and their respective application protocols are illustrated in Figure 1. We administered TibNS using a constant current stimulator with stimulus isolation unit (Grass S48; Warwick, RI, USA). We placed the active lead electrode 1 cm posterior to the right medial malleolus, and the reference electrode 10 cm proximal to the active electrode along the tibial shaft (37).

The stimulation parameters for both intermittent and continuous TibNS consisted of trains of square wave pulses delivered at 30 Hz with a pulse duration of 1 ms. For the intermittent TibNS, stimulus trains were delivered with an on:off duty cycle of 600:400 ms. The intermittent TibNS was delivered for 60 min. To achieve the same total number of stimuli (64,800 pulses), the time of the continuous TibNS was adjusted to 36 min. We delivered TibNS at an intensity of 110% of AH motor threshold. These stimulation parameters are similar to the ones used in previous studies designed to investigate changes in corticospinal excitability following peripheral nerve stimulation in the upper (1, 2, 18) and lower extremity (38). During the period of the TibNS, participants read material of their own choice. Regardless of the stimulation pattern (intermittent or continuous), we maintained the time between pre- and post-TibNS testing in both visits to 60 min. During TibNS, participants were asked to rate their perceived level of stimulation intensity every 5 min on a scale from 0 to 10. In cases when the participant's initial rating changed, the stimulation intensity was adjusted to ensure that they perceived the same level of intensity during the period of TibNS.

### Data analysis

All data analysis procedures were performed by an examiner (GE) who was blinded to the stimulation type and whether the data were from pre- or post-TibNS measures of corticospinal excitability. We used custom-written MATLAB routines to analyze the MEP responses. In each trial, we calculated the average of a 100-ms window of rectified baseline EMG 50 ms prior to the TMS pulse. We defined MEP latency as the time at which the EMG signal following the TMS pulse exceeded a threshold calculated as 2 standard deviations above the mean of the baseline EMG activity and remained beyond this threshold for at least 2 milliseconds (39, 40). For the MEP amplitude calculation, we first plotted the raw EMG activity during all individual TMS trials overlaid on the same graph. We then identified the characteristic biphasic MEP waveform and used the same peaks of this waveform to determine the peak-to-peak MEP amplitude in each individual trial.

We generated the SR curves of each muscle pre- and post-TibNS using the averaged peak-to-peak MEP amplitude values plotted against stimulus intensity (%MSO). We used non-linear curve fitting for each SR curve using a 3-parameter sigmoid function estimated by the Boltzmann equation (41–44), as outlined below:

$$MEP\left(S\right)=\frac{\mathrm{MEPmax}}{1+ex\;p^{m\left(S50\;-\;S\right)}\;}$$

*MEPmax* is the maximum MEP amplitude estimated by the function and is thought to reflect the overall net effect of the total excitatory and inhibitory elements of the corticospinal pathway (42); *S50* is the %MSO at which the MEP amplitude reached 50% of the MEPmax and is also estimated by the function, while *m* is the slope of the SR curve, and *S* is the stimulation intensity (%MSO). We performed the curve fitting procedures in MATLAB using the Levenberg-Marquardt method in the curve fitting toolbox (41–43). Since the change in the slope of the SR curve is expected to occur at the S50, we calculated the peak slope of the tangent line at S50 defined by the component *k* using the following formula (41):

$$k=\;\frac{m\;\times \mathrm{MEPmax}}{4\;}$$

We assessed the goodness-of-fit of the Boltzamnn fit using *R*^2^, and accepted fits with *R*^2^ ≥ 0.80, which would represent a good fit in accordance with previous literature (41, 43, 44). The peak slope (*k*) of the SR curve reflects the steepness (gain) of the function, providing a general measure of corticospinal excitability (43–45). We also calculated the area under curve (AUC) of the SR curve, which reflects the sum of total corticospinal output over a range of TMS intensities (46).

### Statistical analysis

We performed all statistical analyses with SPSS (Version 27.0; IBM Corp., Armonk, NY); we assessed statistical significance at an alpha of 0.05. To ensure the appropriateness of parametric testing, we first examined the experimental data for normality using the Shapiro-Wilk test, and log-transformed the data if necessary. To compare the effects of intermittent vs. continuous TibNS, we compared peak slope, S50, MEPmax, and AUC from the PFM SR curves using a 2 × 2 repeated measures analyses of variance (ANOVA) across *time* (pre vs. post TibNS) and *stimulation* (intermittent vs. continuous). We conducted the same analysis for the AH SR curve parameters. We also reported the partial eta squared (*η*^2^) and Cohen's d effect size of the ANOVAs. If we observed a statistically significant time x stimulation interaction effect, we planned *post hoc* pairwise tests comparing pre- vs. post- continuous TibNS and pre- vs. post-intermittent TibNS, assessed at a Bonferroni-corrected alpha ≤0.025.

In order to determine the consistency of TibNS intensity across test conditions, we used paired samples t-tests to compare stimulus current intensities during continuous and intermittent stimulation. We also used paired samples t-tests to compare the MVC values obtained on Visit 1 and Visit 2 for both AH and PFM in order to determine whether participants achieved comparable levels of attempted MVCs across both visits.

## Results

**Figure 2:**
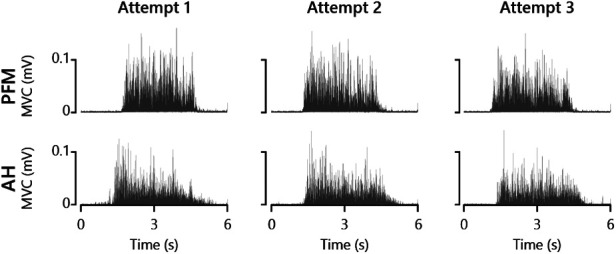
Attempted maximal voluntary contractions (MVC) of PFM and AH from an exemplary participant.

AH MEPs were elicited in all participants. PFM MEPs were obtained in 18 out of 20 participants; in two individuals, we could not elicit any MEP responses from the PFM. The mean AH MEP latency was 43 ms (SD 4) and the mean PFM MEP latency was 29 ms (SD 4). Average MVC values for AH and PFM were 0.030 mV (SD 0.05) and 0.035 mV (SD 0.02), respectively. There were no significant differences in MVC values between both testing visits in either AH (*t* = 0.41, *p* = 0.68) or PFM (*t* = 1.79, *p* = 0.09). Figure 2 shows MVC attempts obtained in both AH and PFM in an exemplary participant.

Mean current intensity during intermittent and continuous TibNS were 28 mA (SD 5) and 27 mA (SD 5), respectively, with no significant difference between stimulation condition (*t* = 1.08, *p* = 0.29). The average change in current intensity during the intermittent stimulation protocol was 2.4 mA ranging between 0 and 9 mA, while during the continuous stimulation it was 1.8 mA and ranged between 0 and 10 mA.

### TMS navigation

**Figure 3:**
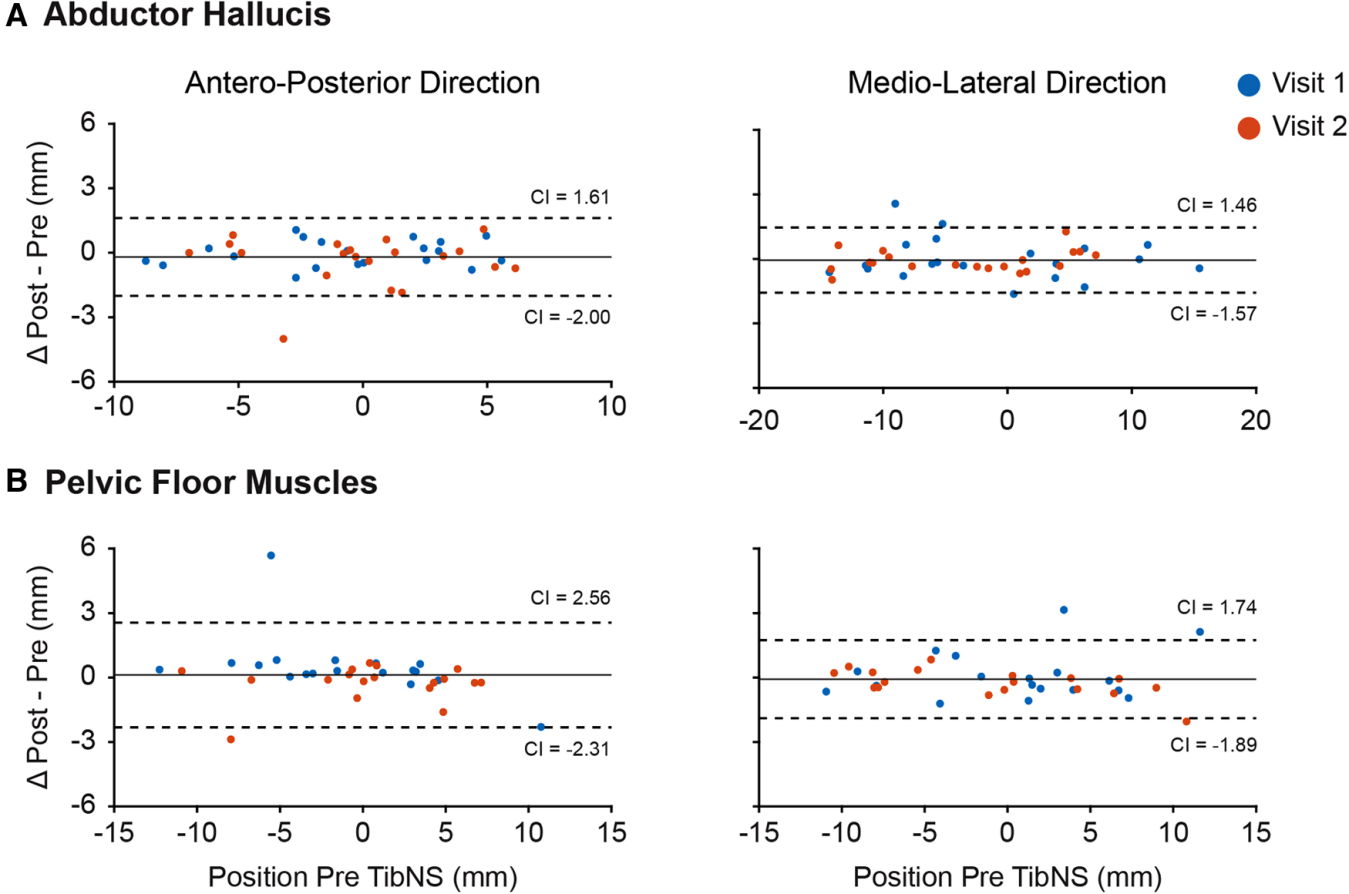
Bland-Altman plot of coil position in antero-posterior and medio-lateral planes during TMS assessment of abductor hallucis (**A**) and pelvic floor muscles (**B**). Each point represents the difference for one participant. The dashed lines represent the 95% confidence intervals (CI), the solid line represents the mean difference of all participants.

The Bland-Altman plots in Figure 3 indicate that we were generally able to maintain consistency in the TMS coil placement in relation to the hot spot pre- and post-TibNS. These plots represent the mean difference in coil positions in antero-posterior and medio-lateral directions pre and post TibNS. Overall, the mean position error of the TMS coil (relative to the defined hot spot location) was less than 5 mm.

### SR curve parameters

**Figure 4:**
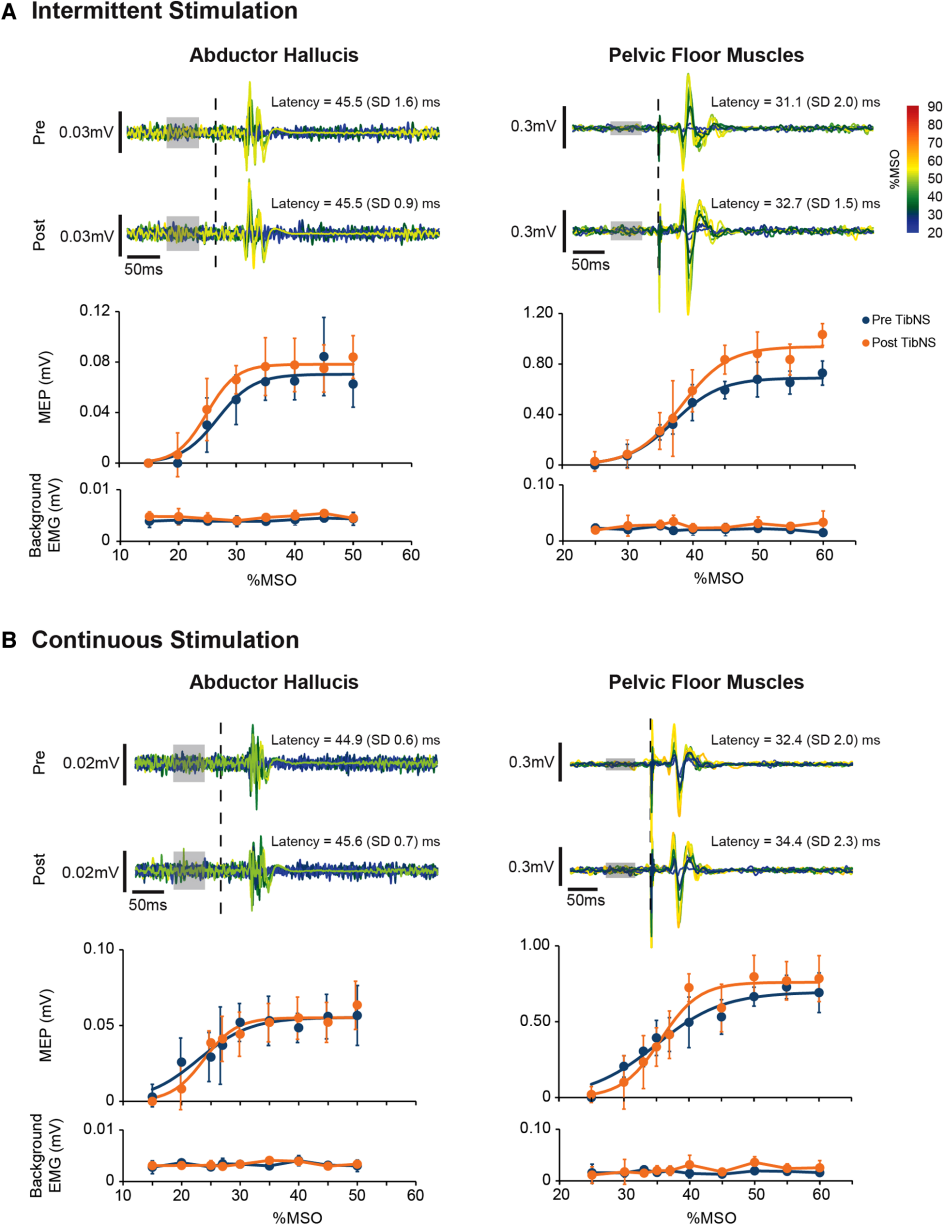
Motor-evoked potentials (MEP) in the abductor hallucis and pelvic floor muscles from an exemplary participant pre and post intermittent (**A**) and continuous (**B**) TibNS. Top panels represent superimposed raw MEP responses to incrementing TMS intensities. The intensity of TMS stimuli [% maximum stimulator output (MSO)] are rainbow color-coded. The time of TMS pulse delivery is indicated by the dotted line. Background EMG used to calculate MEP threshold is indicated by the gray boxes. Bottom panels represent the MEP SR curves pre-and post-TibNS. The mean MEP response at each TMS intensity at pre- and post-TibNS (blue and orange dots, respectively), are plotted with error bars representing standard deviations. Solid lines represent the fitted Boltzmann sigmoidal function. Background EMG is also plotted underneath the SR curves with error bars representing standard deviation to show consistency of background EMG activity between pre- and post-TibNS as well as across different TMS intensities.

The Boltzmann function provided a good fit for AH and PFM SR curves in all participants across both days of testing for both pre-TibNS (mean ± SD coefficient of determination, AH: *R*^2^ = 0.96 ± 0.03; PFM: *R*^2^ = 0.94 ± 0.05) and post-TibNS (AH: *R*^2^ = 0.96 ± 0.03; PFM: *R*^2^ = 0.94 ± 0.03) assessments. Figure 4 provides an example of the raw MEP profiles and the SR curves pre vs. post continuous and intermittent TibNS for AH and PFM from a single participant.

**Figure 5:**
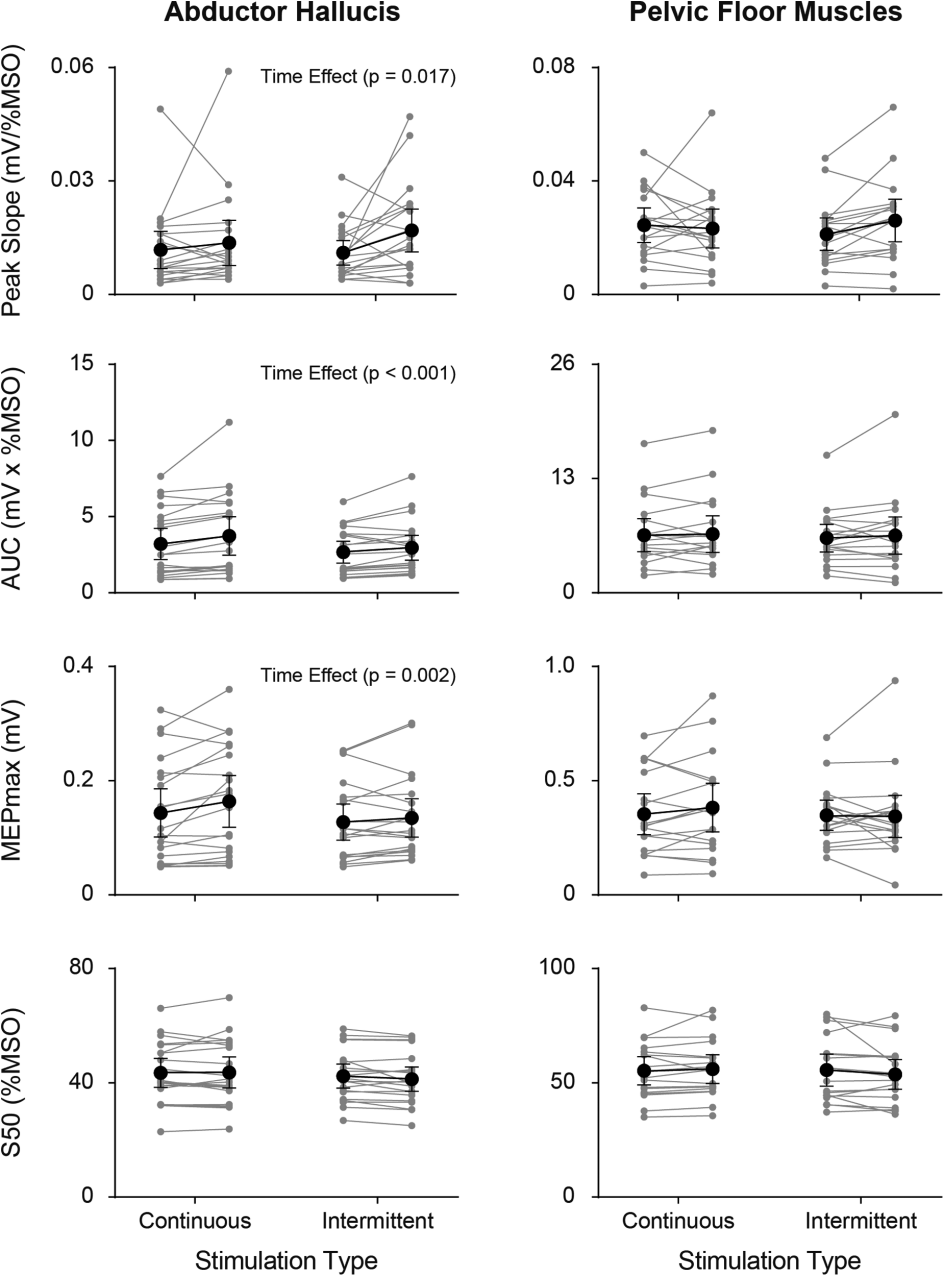
Individual (grey symbols) and mean group (black symbols) values of each SR curve parameter (peak slope, AUC, MEPmax, and S50) from the AH (left panel) and PFM (right panel) pre- vs. post-TibNS. Data from continuous TibNS are plotted on the left side of the graphs and data from intermittent TibNS are plotted on the right. Error bars represent 95% confidence intervals.

SR curve parameters (peak slope, AUC, MEPmax, and S50) pre- and post- continuous and intermittent TibNS are plotted in Figure 5. For the AH muscle, there was a significant main effect of time (pre- vs. post-TibNS) on peak slope [*F* (1,20) = 6.89, *p* = 0.017, *η*^2^ = 0.27, *d* = 0.61], AUC [*F* (1,20) = 25.48, *p* < 0.001, *η*^2^ = 0.57, *d* = 1.15] and MEPmax [*F* (1,20) = 13.50, *p* = 0.002, *η*^2^ = 0.42, *d* = 0.84], but not on S50. There was no time×stimulation interaction effect. For the PFM, there were no significant effects in any of the SR curve parameters.

## Discussion

In this study, we compared the changes in corticospinal excitability of a target (AH) and non-target muscles (PFM) following TibNS. We also compared the effects of applying TibNS using continuous vs. intermittent patterns on corticospinal excitability to these muscles. Our results indicate that peak slope, AUC, and MEPmax of the SR curves of the AH muscle, but not the PFM, increased after TibNS. We did not observe any effect of stimulation pattern.

### Targeted change in corticospinal excitability

Many studies have demonstrated the neuromodulatory effects of peripheral nerve stimulation on the excitability of corticospinal pathways in humans (1, 4, 9, 14, 15, 18, 19, 47–50). Studies of upper limb muscles show a focal, targeted effect of peripheral nerve stimulation on corticospinal excitability, while effects in the lower limb seem to be more diffuse (19, 38, 49). This has been explained by differing functional roles of the upper compared to the lower limbs; afferent projections from the hand may be more specific given the precision required for skillful movements compared to the more integrated afferent regulation from the lower limb needed to regulate gross motor requirements of balance and locomotion (19). However, the diffuse effects of peripheral neuromodulation on corticospinal excitability do not seem to be consistent across nerves of the lower limb. While there is evidence that continuous stimulation of the common peroneal nerve can result in diffuse effects to muscles with different innervation (i.e., vastus medialis and soleus) (19), similar stimulation delivered to the tibial nerve at the popliteal fossa failed to affect homonymous corticospinal connections to the soleus muscle (51). Rather, changes in excitability following a bout of tibial nerve stimulation may be limited to spinal reflex pathways (soleus H-reflex), and only when it was combined with voluntary plantarflexion exercise (51).

In the present study, we stimulated the tibial nerve further distally, behind the medial malleolus, and found evidence supporting enhanced homonymous corticospinal excitability to the abductor hallucis muscle. There may be multiple reasons for our divergent results from Lagerquist et al. (2012). Lagerquist et al. (2012) tested motor-evoked potentials at a single stimulus intensity (120% of active motor threshold) whereas we characterized the full stimulus-response profile to the AH, so it is possible that they may have missed an overall shift in modulation of corticospinal excitability. Indeed, although we found an overall statistically significant increase in corticospinal excitability (peak slope) to the AH, the overall effect sizes were moderate and inspection of individual subject data (Figure 5) indicates variability in the pre-post changes in these parameters across participants. The respective functional roles of the AH and soleus muscles may also be a factor. The contribution of reflex pathways from the soleus in gait and balance function has been well characterized (52–54), while sensory input from intrinsic foot muscles, such as the AH, is thought to help regulate foot posture during standing and gait (55, 56). However, whereas voluntary ankle plantarflexion can be performed with ease, many of our participants expressed difficulty performing flexion and especially abduction of the first metatarsophalangeal joint (the action of AH), an observation also noted by others (57, 58). We did not examine any other muscles of the lower limb, so there is further opportunity to characterize the distribution of neuromodulation across different muscles and peripheral nerves of the lower limb.

Given the key role of the PFM in different functional tasks (e.g., to maintain continence in instances of high intra-abdominal pressure, such as running or jumping), as well as the fact that the segmental innervation of the PFM overlaps with that of the tibial nerve, we anticipated possible diffuse effects of TibNS extending to this axial muscle. However, we did not find evidence that the afferent input from TibNS can increase the corticospinal excitability to the PFM. We are unable to rule out whether any stimulation-induced effects might have occurred outside the corticospinal tract. For example, it is possible that the TibNS modulated transmission along spinal reflex pathways to the PFM that we would not be able to detect with TMS. Reflex responses in the external anal sphincter as well as the external urethral sphincter can be elicited by stimulation of the tibial nerve behind the medial malleolus (59, 60). Response latencies of this reflex arc did not differ between uninjured controls and subjects with central nervous system lesions resulting in spastic paralysis, suggesting a spinal origin of this cutaneous reflex pathway (60). Future studies could explore the possibility that there may be changes in the excitability along spinal reflex circuitry from the tibial nerve to PFM motor pools following TibNS.

### Lack of effect of stimulation pattern

We evaluated the effects of the pattern of electrical stimulation (intermittent vs. continuous) while controlling for the other stimulation parameters of frequency, pulse duration, total number of pulses delivered, and intensity. Ishibashi et al. (2021) reported that under controlled conditions (equal frequency, intensity, and total number of pulses), intermittent stimulation is likely to increase, while continuous stimulation is likely to suppress the excitability of M1 and S1 (18). They speculate that continuous stimulation might have resulted in habituation of the primary somatosensory cortex (18), where the consistency of the stimulation represents little information relevant to movement, and instead, causes a gating phenomenon of the somatosensory system leading to suppression of S1 (4, 18). The intermittent or motor stimulation, on the other hand, supplies the somatosensory cortex with afferent signals not only from the nerve stimulation, but also from the contracting muscle itself (4). Therefore, this pattern of stimulation could be construed as more relevant and functional by the CNS (4, 18).

In our study, we found no difference between the effects of intermittent and continuous TibNS; both patterns of stimulation were associated with increases in AH corticospinal excitability. One possible explanation could be the fact that during our experimental protocol, we instructed our participants to pay close attention to TibNS by asking them to rate the intensity of the stimulation every five minutes. If the level of perceived intensity decreased, we adjusted the current intensity accordingly. Perhaps through this constant cognitive attention to the stimulation we may have inadvertently suppressed the level of stimulus habituation in our participants. Furthermore, TibNS, as used clinically to manage the symptoms of lower urinary tract dysfunction, is delivered continuously and at sub-motor threshold (sensory) levels of stimulation (10, 11); future studies should consider how a more clinically-applied mode of stimulation may differently affect changes in corticospinal excitability.

Another possible reason could be that we used a longer pulse width duration (1 ms) compared to the experiments by Ishibashi et al. (18) (0.2 ms) and Schabrun et al. (4) (0.1 ms). It has been reported that longer pulse durations (≥0.5 ms) generate greater synaptic recruitment of spinal motoneurons *via* reflexive pathways compared to shorter pulse durations (61–63). Thus, our stimulation protocol might have recruited a larger proportion of motor units through reflexive pathways, while the stimulation parameters in the aforementioned studies may have activated more motor units through direct activation of efferent axons. Although their stimulation intensities were high enough to activate a large number of afferents, the alpha motoneurons might have been less responsive to the synaptic input *via* reflex pathways compared to our stimulation protocol. The reason behind this is due to the large amount of antidromic propagation of action potentials making the membrane potentials of the alpha motoneurons more refractory. Moreover, evidence shows that longer pulse durations recruit motoneurons in accordance with the Henneman's size principle, while the shorter pulse durations recruit motoneurons in a more random, non-physiological order (61–63). Therefore, our continuous stimulation, with 1-ms pulse durations, may have elicited a more physiologically typical contraction of the AH, which could have potentially been more difficult to habituate to. These are, however, speculations that have to be supported by future experiments investigating the integrated effects of pulse duration and stimulation pattern on corticospinal excitability.

### Methodological considerations

There is a lack of standardized protocols to measure corticospinal excitability of the PFM. Although SR curves have been regarded as a relatively stable characteristic of the corticospinal projections and a reliable measure of corticospinal excitability, they have only been extensively characterized for the distal muscles of the limbs. Some studies that examined the reliability of the SR curves in the proximal muscles of the upper extremity suggest they are less reliable (46, 64) compared to measures obtained in intrinsic hand muscles (41, 44) and tibialis anterior (65). Perhaps, there could be inherent differences in the input-output properties of corticospinal projections between muscles designed for a greater level of dexterity compared to those used for postural stability. Axial muscles have a smaller amount of direct pyramidal tract projections (66–68) and may rely more on the input from extrapyramidal pathways compared to the distal muscles of the arms and legs (68). In fact, there are no studies, to the best of our knowledge, that characterize and assess the reliability of the SR curve parameters in PFM. Nevertheless, we note in our data that the Boltzmann function provided excellent fits for SR curves of both AH and PFM (*R*^2^ ≥ 0.94).

Previous studies have suggested that comfort and alertness can affect the variability of the MEPs (69, 70). Our experimental procedures were approximately 4–5 h long, and during such an extended time period, it is possible our participants' level of alertness or fluctuation in motivation could have impacted cortical excitability.

## Conclusion

Our results demonstrate that both intermittent and continuous TibNS may increase the corticospinal excitability of the AH, but not of the PFM. Despite the fact that our study did not show corticospinal modulation of the PFM, additional experiments are required to identify whether changes in spinal pathways affecting the PFM could be observed following TibNS. Furthermore, given the range of stimulation parameters used across laboratory and clinical investigations, future investigations are warranted to understand how pattern and intensity, among other stimulation variables could explain the neurophysiological mechanisms subserving the somatic and/or autonomic effects of various neuromodulation therapies.

## Data Availability

The original contributions presented in the study are included in the article/Supplementary Material, further inquiries can be directed to the corresponding author/s.
